# A Rare Association of Autoimmune Hypophysitis With Seronegative Rheumatoid Arthritis: A Case Report

**DOI:** 10.7759/cureus.59167

**Published:** 2024-04-27

**Authors:** Amna Kamran, Ahmed Ghazy, Narine Misakyan, Tehreem Fatima, Nazish Najeeb

**Affiliations:** 1 Internal Medicine, Capital Health Regional Medical Center, Trenton, USA; 2 Internal Medicine, King Edward Medical University, Mayo Hospital, Lahore, PAK

**Keywords:** endocrine, disease-modifying antirheumatic drugs (dmards), diabetes insipidus, xanthomatous hypophysitis, autoimmune, seronegative rheumatoid arthritis, autoimmune hypophysitis

## Abstract

Autoimmune hypophysitis (AH) is an uncommon condition where there is inflammation of the pituitary gland which leads to hormonal imbalances. It is often associated with autoimmune diseases; however, a case is yet to be reported with an association of AH with seronegative rheumatoid arthritis (RA). We present a case of a 45-year-old female who complained of polyuria/polydipsia and rapid weight gain. An MRI of the head revealed enlargement of the pituitary gland, concerning for AH. Although she was initially treated for diabetes insipidus, she began reporting new complaints of joint pains and morning stiffness. She was clinically diagnosed with seronegative RA and improved with a trial of hydroxychloroquine. A repeat MRI showed improvement in the abnormal pituitary findings, and the patient was closely monitored with a multidisciplinary approach. Diagnosing and managing patients with AH are topics that are still being explored and researched as it is a relatively rare pathology. Consequently, we found the need to discuss the relationship of AH with seronegative RA and delve into the various diagnostic and treatment approaches.

## Introduction

Autoimmune hypophysitis (AH) is a rare and often underdiagnosed pituitary disorder characterized by inflammation of the pituitary gland caused by the immune system attacking the body’s tissues [1,2]. Rheumatoid arthritis (RA) [3] is a systemic autoimmune disorder that affects multiple organs and tissues, including the joints. Although AH and RA may coexist, cases of concomitant AH and RA are exceedingly rare. In this case report, we present a patient with AH associated with seronegative RA and discuss the clinical presentation, diagnostic workup, and management of this uncommon condition.

## Case presentation

**Figure 1:**
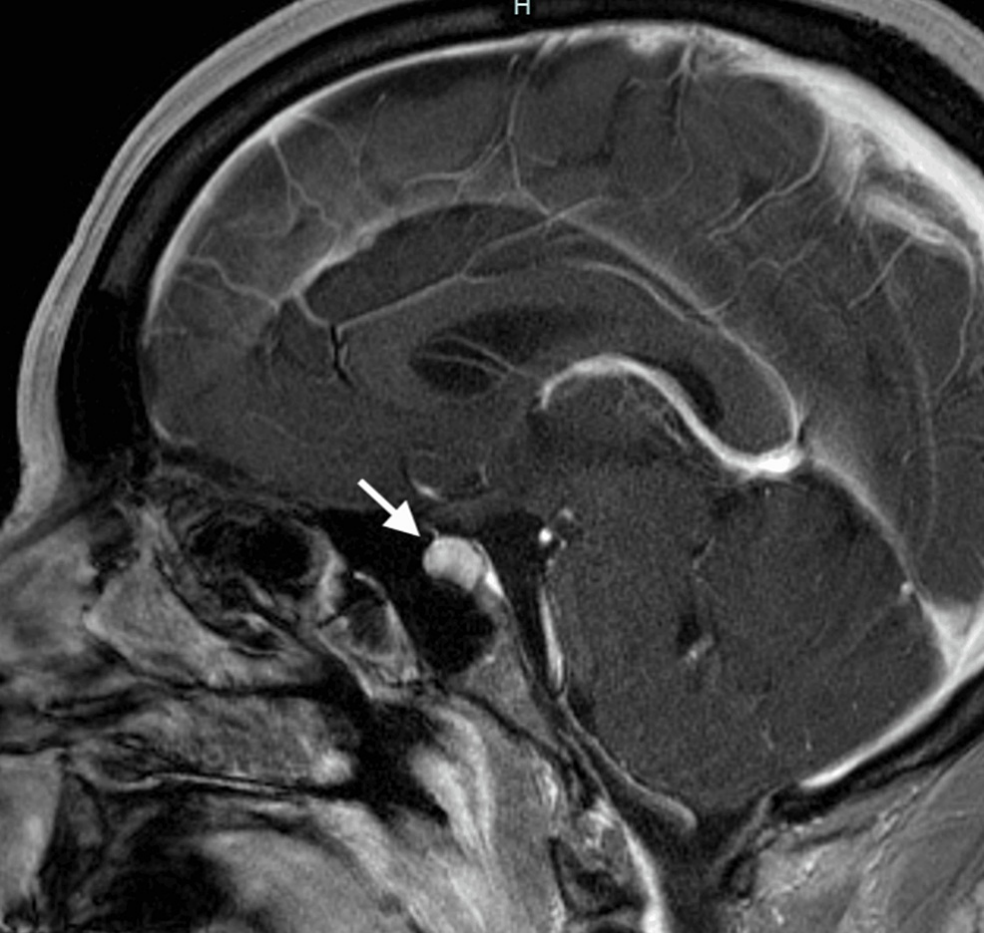
MRI of the brain and pituitary showing prominence of the pituitary gland with heterogenous enhancement.

A 45-year-old woman visited the emergency department with excessive thirst, increased water intake, and rapid weight gain. An MRI of her brain revealed slight enlargement and enhancement of the pituitary gland and infundibulum (Figure 1). She was diagnosed with diabetes insipidus (DI) based on her symptoms (polyuria and polydipsia), lab work (high plasma osmolarity and low urine osmolarity), and MRI findings. The patient was prescribed desmopressin tablet 0.2 mg daily and was referred to the endocrinologist.

During the follow-up, the patient reported improved polyuria and thirst but developed headaches, memory loss, fatigue, and discomfort in her fingers and legs. She also experienced irregular periods, nipple discharge, photosensitivity, extremely dry eyes and mouth, and cystic acne. Blood tests revealed a high prolactin level of 62.6 ng/mL and low follicle-stimulating hormone and luteinizing hormone levels. Cabergoline 0.5 mg twice weekly was prescribed, and a referral to a rheumatologist was made due to suspicion of AH. At the rheumatology visit, multiple blood tests were ordered with the results shown in Table 1.

**Table 1:** Laboratory test results. ESR = erythrocyte sedimentation rate; CRP = C-reactive protein; ACTH = adrenocorticotropic hormone; ACE = angiotensin-converting enzyme; FSH = follicle-stimulating hormone; LH = luteinizing hormone; TSH = thyroid-stimulating hormone; IGF = insulin-like growth factor; P-ANCA = perinuclear anti-neutrophil cytoplasmic antibodies; C-ANCA = antineutrophil cytoplasmic antibodies; SCL= scleroderma

Laboratory test	Result	Reference range
Serum osmolarity	301 mOsm/kg H_2_O	275–295
Urine osmolarity	108 mOsm/kg H_2_O	50–1,400
Prolactin	62.6 ng/mL	3.0–30.0
FSH	8.7 mIU/mL	3.5–12.5
LH	5.6 mIU/mL	2.4–12.6
ACTH	15 pg/mL	6–50
Cortisol	16.9 µg/dL	4.0–22.0
TSH	2.910 µIU/mL	0.450–4.500
IGF	165 ng/mL	74–239
Rheumatoid factor, qualitative	Negative	Negative
Anti-cyclic citrullinated peptide IgG/IgA	18 units	0–19
14-3-3 eta protein	<0.2 ng/mL	<0.2
Antinuclear antibodies, IFA	Negative	Negative
Anti-dsDNA antibodies	1 IU/ml	<5
Sjogren antibody panel (anti-SSA/SSB)	<0.2 AI	0–0.9
SCL-70 antibodies	<0.2 AI	0–0.9
Smith ENA + ribonucleoprotein ENA	<0.2 AI	0–0.9
C-ANCA	<1:20	<1:20
P-ANCA	<1:20	<1:20
Anti-centromere antibodies	<0.2 AI	0–0.9
ACE serum level	38 U/L	14–82
Gamma-glutamyltransferase	14U/L	5–36
Actin smooth muscle IgG Ab	9 units	0–19
Anti-mitochondrial (M2) Ab	<20 units	0–20
Beta-2 glycoprotein 1 IgA + IgG + IgM Ab	Negative	Negative
Plasma lupus anticoagulant	Negative	Negative
Anticardiolipin IgG + IgM + IgA Ab	Negative	Negative
Vitamin D 25-hydroxylase	38	>30
Vitamin B12	872	232–1,245
CRP	<5	<8
ESR	53	<20

**Figure 2:**
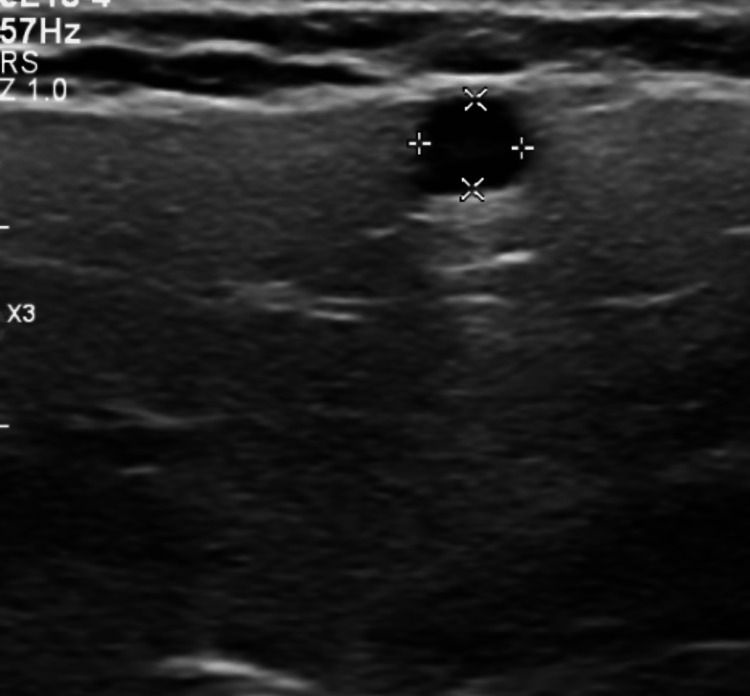
Ultrasound of the parotid gland showing the left parotid cyst.

**Figure 3:**
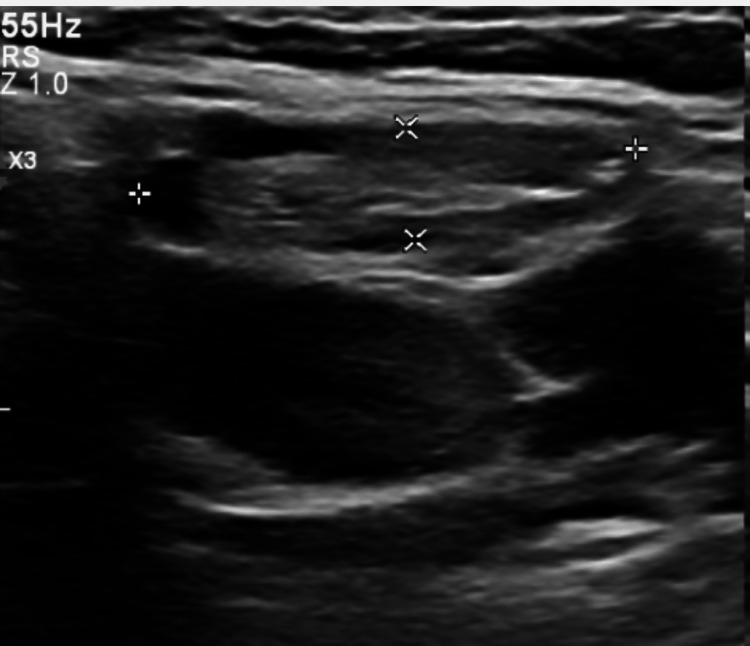
Ultrasound of the right parotid gland.

The chest X-ray was unremarkable. An ultrasound of theparotid gland was done due to the patient’s complaints of severely dry mouth, which revealed a 4 mm cyst within the left parotid gland (Figure 2) and a fatty lymph node adjacent to the right parotid gland measuring 21 mm (Figure 3).

The patient was prescribed celecoxib 100 mg orally twice daily and scheduled for a repeat MRI in three months. Neurosurgery was consulted with no surgical intervention or biopsy recommended. The patient continued to take cabergoline, and her prolactin level normalized on repeat blood work after six months, decreasing from 62.6 ng/mL to 2.6 ng/mL.

After four weeks, the patient reported increased headaches, fatigue, and difficulty functioning, along with severe prolonged morning stiffness in her fingers, which improved after usage. Due to a high suspicion of seronegative RA and AH, she was prescribed hydroxychloroquine 200 mg tablets (one tablet a day for three days, and then one tablet twice a day) and started on methotrexate 10 mg once a week with daily folic acid, which was later increased to 12.5 mg weekly.

**Figure 4:**
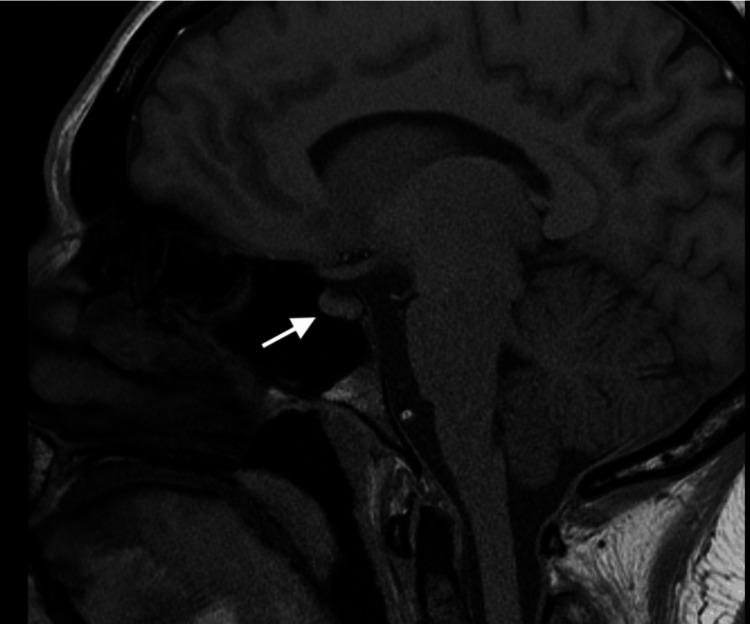
MRI of the brain and pituitary showing stable nodular thickening of the pituitary infundibulum involving the superior portion.

A follow-up MRI in three months (Figure 4) showed stability and some improvement in the imaging when discussed with the neurosurgeon. The patient reported improvement in joint discomfort, confirming the suspicion of seronegative RA. However, cognitive difficulties persisted. A referral to a neurologist was made for cognitive issues. Regular follow-up appointments were maintained with the endocrinologist, rheumatologist, and neurologist.

## Discussion

AH [1] is a rare disease with an incidence of one in nine million/year [4,5] according to published studies. Since the first description by Goudie and Pinkerton [6] of lymphocytic AH in 1962, hundreds of cases have been recognized as per a recent meta-analysis. However, a case is yet to be reported of AH in association with seronegative RA.

In this case, a 45-year-old female presented with polyuria and galactorrhea with MRI findings of an enlarged pituitary with homogenous enhancement and a thickened stalk. The patient also reported persistent joint pains and morning stiffness with clinical evidence of synovitis and was started on methotrexate and hydroxychloroquine with symptomatic improvement. Overall, 6%-60% of reported cases have had associations with autoimmune conditions, with the most common ones being Hashimoto thyroiditis and Graves’ disease. The autoimmune workup proved to be unremarkable apart from an elevated erythrocyte sedimentation rate. Given her typical arthritic presentation and joint synovitis, she was diagnosed with seronegative rheumatoid arthritis and was treated with disease-modifying antirheumatic drugs with notable improvement. A rare case also reported an association of RA with biopsy-proven xanthomatous hypophysitis (XH) [3], which is a subtype of AH, in which the importance of suspecting XH in those who have pre-existing autoimmune conditions was discussed.

Further workup was also consistent with DI and the patient was started on desmopressin. No other hormonal irregularities were noted upon review. In most reported cases of AH, headaches and polyuria/polydipsia are common presenting complaints. However, typically, AH cases depict hormonal imbalances in relation to the thyroid, adrenal, and gonadal axes, which were absent in this case.

Studies have shown that glucocorticoid-sparing agents such as methotrexate and azathioprine may improve AH findings [7]. Cases have also been published where the usual standard of treatment has been with steroids if the presentation includes symptoms secondary to compression. We opted against steroids as there was no visual impairment and we noted improvement without it. Given the extensive side effect profile, including high relapse rates, our case so far has not warranted treatment with steroids.

The diagnostic approach to such cases is controversial. Biopsy/surgery is still set as the gold standard for diagnosis; however, given its invasive nature, a more conservative approach is usually chosen despite its ambiguity [1,8]. We adopted a similar approach in our case, where a biopsy was deferred and monitoring with imaging was preferred. Gutenberg proposed a new radiologic score for the diagnostic MRI findings to distinguish between AH and pituitary adenomas. Using that scale, this case landed a score of congruous with AH. Several case reports have been published deeming a high enough clinical suspicion to be good enough to determine the diagnosis and management. However, as the clinical presentations are quite overlapping, narrowing down to a single diagnosis can be challenging.

## Conclusions

To our knowledge, no such case has been published regarding the association of seronegative RA with AH. This case seems to be extremely uncommon and rare in its overall findings. Discussing such cases helps add to the already existing pool of reported cases to enhance our knowledge about the etiology and possible management avenues. It is especially imperative to be mindful of atypical presentations such as ours so that further research can be done regarding varying associations and risk factors.
